# Rapid Onset of Motor Deficits in a Mouse Model of Spinocerebellar Ataxia Type 6 Precedes Late Cerebellar Degeneration^1,2,3^

**DOI:** 10.1523/ENEURO.0094-15.2015

**Published:** 2015-12-26

**Authors:** Sriram Jayabal, Lovisa Ljungberg, Thomas Erwes, Alexander Cormier, Sabrina Quilez, Sara El Jaouhari, Alanna J. Watt

**Affiliations:** Department of Biology, McGill University, Montreal, Quebec, Canada

**Keywords:** ataxia: behavioral assays, cerebellum, neurodegeneration, Purkinje cell

## Abstract

Spinocerebellar ataxia type 6 (SCA6) is an autosomal-dominant cerebellar ataxia that has been associated with loss of cerebellar Purkinje cells. Disease onset is typically at midlife, although it can vary widely from late teens to old age in SCA6 patients. Our study focused on an SCA6 knock-in mouse model with a hyper-expanded (84X) CAG repeat expansion that displays midlife-onset motor deficits at ∼7 months old, reminiscent of midlife-onset symptoms in SCA6 patients, although a detailed phenotypic analysis of these mice has not yet been reported. Here, we characterize the onset of motor deficits in SCA6^84Q^ mice using a battery of behavioral assays to test for impairments in motor coordination, balance, and gait. We found that these mice performed normally on these assays up to and including at 6 months, but motor impairment was detected at 7 months with all motor coordination assays used, suggesting that motor deficits emerge rapidly during a narrow age window in SCA6^84Q^ mice. In contrast to what is seen in SCA6 patients, the decrease in motor coordination was observed without alterations in gait. No loss of cerebellar Purkinje cells or striatal neurons were observed at 7 months, the age at which motor deficits were first detected, but significant Purkinje cell loss was observed in 2-year-old SCA6^84Q^ mice, arguing that Purkinje cell death does not significantly contribute to the early stages of SCA6.

## Significance Statement

We confirm that disease onset in an 84Q-hyperexpanded polyglutamine mouse model of spinocerebellar ataxia type 6 (SCA6) occurs at 7 months of age, which is in agreement with a previous study by Watase et al. (2008). We characterize disease onset more precisely using a barrage of behavioral tests at multiple ages, and identify that motor coordination abnormalities emerge in a narrow time window between 6 and 7 months, in contrast with the variable age of onset observed in human patients. We find that Purkinje cell degeneration occurs in this SCA6 mouse model at 2 years, nearly 1.5 years after the onset of motor deficits, demonstrating that Purkinje cell loss is not necessary for early SCA6 disease symptoms.

## Introduction

Spinocerebellar ataxia type 6 (SCA6) is an autosomal-dominant neurodegenerative disease that leads to progressive ataxia of the limbs and gait abnormalities, and is one of the most common of the spinocerebellar ataxias (Ashizawa et al., 2013). SCA6 is caused by a CAG-repeat expansion in the gene *CACNA1A* encoding the α1A-subunit of voltage-dependent P/Q-type calcium channel, causing a polyglutamine (poly-Q) expansion (Zhuchenko et al., 1997). P/Q channels are widely expressed in the brain, including in cerebellar Purkinje cells (Westenbroek et al., 1995; Craig et al., 1998), which undergo degeneration in SCA6 (Yang et al., 2000). In patients, SCA6 symptoms typically present in midlife, with an average onset of ataxic symptoms at ∼40-50 years of age (Matsumura et al., 1997; van de Warrenburg et al., 2002; Ashizawa et al., 2013), although disease onset has been observed across a wide range of ages, from late teens to old age (Yabe et al., 1998).

The size of the repeat expansion that gives rise to SCA6 is short compared with other triplet-repeat diseases (Gatchel and Zoghbi, 2005): unaffected individuals have <20 repeats, while pathological repeat length is 20-33 (Yabe et al., 1998; van de Warrenburg et al., 2002; Gatchel and Zoghbi, 2005). Consistent with several other triplet-repeat diseases, there is an inverse relationship between CAG repeat expansion length and age of onset in SCA6: longer repeats are correlated with earlier onset of symptoms (Matsumura et al., 1997; van de Warrenburg et al., 2002; Ashizawa et al., 2013). However, the relationship of repeat length with the age of disease onset is estimated to account for only 52% of the variance in the age of onset of SCA6 (van de Warrenburg et al., 2002), meaning that individuals who have the same repeat length can differ in the age at which they are first affected by SCA6 by decades. We wondered whether similar variability is observed in animal models of SCA6, since this may give insight into the origin of variability of disease onset in human patients.

Several mouse models have been developed for SCA6 that show a broadly similar relationship between repeat length and gene dosage on disease onset and severity as that observed for human patients, although typically shifted toward longer repeat lengths than those observed in human patients. Mice with human-length triplet repeats (SCA6^30Q^) have not been observed to develop motor deficiencies (Watase et al., 2008), while a homozygous knock-in mouse model that harbors a hyperexpanded 84-CAG repeat in the encoding region of the P/Q channel subunit (SCA6^84Q^) displays late-onset motor symptoms similar to human patients: homozygous mice show no motor abnormalities at 3 months but exhibit motor deficits at 7 months (Watase et al., 2008). Furthermore, a mouse with an even longer CAG repeat (SCA6^118Q^) displays motor impairment as early as 6 weeks old (Unno et al., 2012). While Purkinje cell loss has been reported to rapidly follow motor deficits in the SCA6^118Q^ transgenic mouse (detected at 10 weeks; Unno et al., 2012), no Purkinje cell loss has been reported to date in the late onset SCA6^84Q^ mouse (Watase et al., 2008). More recently, mice overexpressing P/Q-type calcium channel C-terminal fragments containing human-length triplet-repeat insertions have been developed that display motor phenotype (Du et al., 2013; Mark et al., 2015).

Since the variable onset of disease symptoms in SCA6 patients is only partially explained by differences in repeat length (van de Warrenburg et al., 2002), we wondered whether variability existed in disease onset in a mouse model of SCA6 as well. Since the SCA6^84Q^ mouse best recapitulates the midlife onset observed in human SCA6 (Watase et al., 2008), we chose to study the onset of motor coordination symptoms in more detail in the SCA6^84Q^ mouse in order to pinpoint the age of onset of disease symptoms more accurately. We assayed motor coordination of SCA6^84Q^ mice at multiple postnatal ages using several motor coordination assays including rotarod, elevated beam, and swimming. We found that motor deficits were detected simultaneously with all motor coordination assays, suggesting that there is a narrow and rapid age of onset in this SCA6^84Q^ mouse model, which is strikingly different from the high variability in the age of onset observed in human patients. Motor coordination deficits occurred in 7-month-old mice without any observable difference in gait or changes in Purkinje cell number or morphology, and gait abnormalities were not found even in 2-year-old mice. Although Purkinje cell degeneration was not observed at 7 months in SCA6^84Q/84Q^ mice, these mice have fewer Purkinje cells than wild-type (WT) mice at 2 years, arguing that although Purkinje cell death may contribute to disease progression in SCA6, it does not significantly contribute to early stages of SCA6.

## Materials and Methods

### Animals

Transgenic SCA6^84Q^ mice were purchased from The Jackson Laboratory (strain B6.129S7-*Cacna1atm3Hzo*/J), and heterozygous mice were bred in order to produce litter-matched male and female transgenic SCA6^84Q^ (homozygous SCA6^84Q/84Q^, and heterozygous SCA6^84Q/+^) and WT mice. At each age, behavioral assays were performed on naive animals with no prior exposure to the assays during a period of 5 consecutive days (for animal numbers at each age, see Table 1), and all data were acquired blind to genotype. Animals were moved from the housing room to the experiment room and allowed 30 min to acclimatize before beginning experiments on each day of testing. Assays were performed in same order, as follows: (1) rotarod; (2) elevated beam assays on days 1–4; (3) swimming; and (4) gait was tested last on day 5 (D5) of testing for 3- to 7-month-old mice. For 1- and 2-year-old mice, gait was tested first on the first day of testing prior to the rotarod assay.

**Table 1:** Sample size for each genotype at each experimental age

Genotype	N for each experimental age						
	3 Months	4 Months	5 Months	6 Months	7 Months	1 Year	2 Years
WT	7	9	8	7	6	8	7
SCA6^84Q/+^	6	9	7	8	5		
SCA6^84Q/84Q^	9	9	8	10	9	8	8

Summary of the number of animals (*N*) for each of three genotypes used at each experimental age (mice were naive at each age without any prior behavioral training).

### Rotarod assay

**Figure 1.**
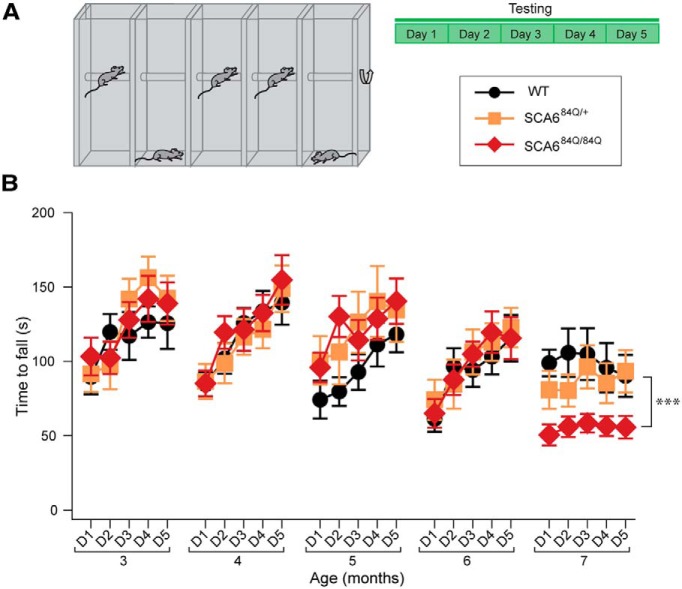
Rotarod deficits at 7 months in SCA6^84Q/84Q^ mice. ***A***, Schematic of experimental paradigm: accelerating rotarod experiments were conducted in four trials per day for 5 days of testing at each age. ***B***, No significant differences on D4 and D5 were observed among SCA6^84Q/84Q^, SCA6^84Q/+^, and WT genotypes at 3, 4, 5, or 6 months old; however, SCA6^84Q/84Q^ mice display poorer performance on rotarod on D4 and D5 at 7 months compared with WT mice (Genotype: *F*_(2,37)_ = 12.19; *p* = 0.0004, one-way ANOVA with *post hoc* Tukey’s test; ***p < 0.0005; *p* > 0.05 where not indicated; *N* = 8 − 10 SCA6^84Q/84Q^ mice depending on age, 5 − 9 SCA6^84Q/+^ mice, and 6 − 9 WT mice (consult Table 1 for sample size at each age).

Animals were placed on a rotarod (Stoelting Europe) using a standard 10-min-long accelerating assay where the rod accelerates from 4 to 40 rpm in the first 5 min and then continues to rotate at 40 rpm for the last 5 min (Carter et al., 2001; Watase et al., 2007; Fig. 1*A*; Movie 1, 2). The latency to fall was recorded for each mouse as a measure of cerebellum-related motor coordination (Watase et al., 2007). Mice performed four trials (T1–T4) per day, and had at least a 15 min resting period between trials, over 5 consecutive days of testing (D1–D5).

**Movie 1.** Rotarod assay. SCA6^84Q/84Q^ mouse (right chamber) spends less time on an accelerating rotating rod compared with the litter-matched WT control mouse (left chamber) at 7 months.

**Movie 2.** Sample rotarod assay (entire trial at high speed). SCA6^84Q/84Q^ mouse (right chamber) spends less time on an accelerating rotating rod compared with the litter-matched WT control mouse (left chamber) at 7 months. Mice are the same as in Movie 1, but the entire trial is shown, at 4× speed.

### Elevated beam assay

**Figure 2.**
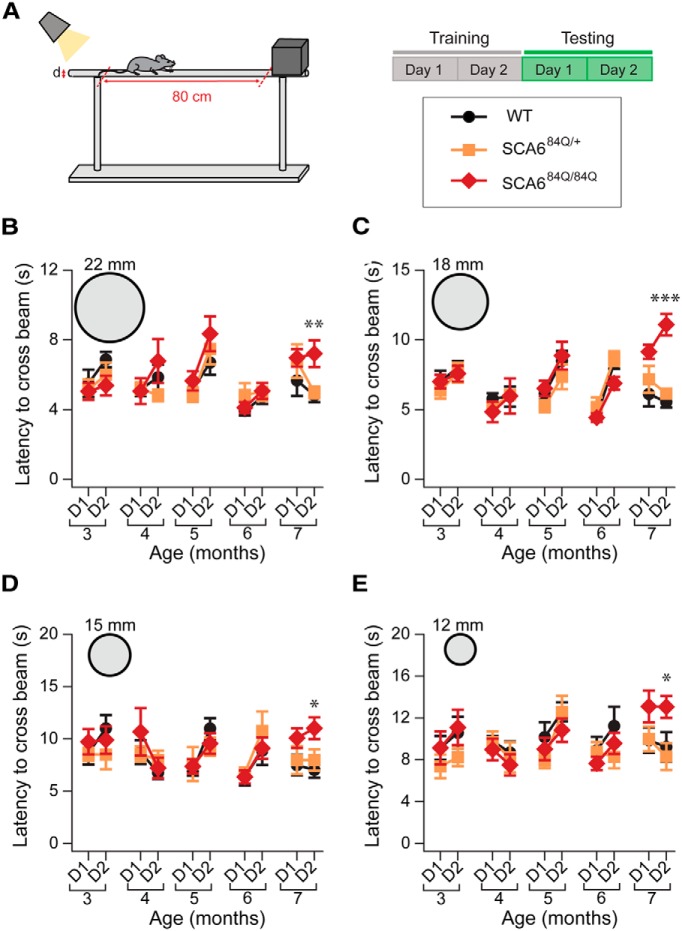
Increased latency on elevated beam at 7 months in SCA6^84Q/84Q^ mice. ***A***, Schematic of experimental design for elevated beam assay. Two days of training were followed by 2 d of testing (D1 and D2 in ***B*−*E***). ***B*−*E***, Latency to cross the beam was measured for each genotype at each age (3, 4, 5, 6, and 7 months) over D1 and D2. SCA6^84Q/84Q^ mice were significantly slower at traversing the beam at 7 months on D2 for the following diameters: ***B***, 22 mm (*F*_(2,17)_ = 7.36; *p* = 0.005); ***C***, 18 mm (*F*_(2,17)_ = 7.46; *p* = 0.005); ***D***, 15 mm (*F*_(2,17)_ = 4.34; *p* = 0.03); and ***E***, 12 mm (*F*_(2,17)_ = 5.27; *p* = 0.017). SCA6^84Q/+^ mice were indistinguishable from WT mice. *p < 0.05, **p < 0.01, ***p < 0.005; *p* > 0.05 where not indicated, one-way ANOVA followed by *post hoc* Tukey’s test; *N* = 8 − 10 SCA6^84Q/84Q^ mice depending on age, 5 − 9 SCA6^84Q/+^ mice, and 6 − 9 WT mice (consult Table 1 for sample size at each age).

Animals walked along a custom-built apparatus consisting of raised round wooden beams (100 cm long), toward a dark escape box, as previously described (Carter et al., 2001; Fig. 2*A*; Movie 3). Bright light shining on the starting point was used as an aversive stimulus to encourage mice to traverse the beam. D1 and D2 were training days, during which mice were trained to cross a beam 22 mm in diameter. On D3 and D4, corresponding to testing D1 and D2, each mouse performed a trial on beams with diameters of 22, 18, 15, and 12 mm, totaling four trials per day. The time taken to traverse 80 cm was recorded, and the number of times the foot of the mouse slipped while crossing the beams was counted during *post hoc* video analysis (for an example of a mouse whose foot slips three times during the assay, see Movie 4).

**Movie 3.** Elevated beam assay. A 7-month-old WT mouse crosses an elevated beam.

**Movie 4.** Elevated beam assay illustrating footslips (in slow motion). An SCA6^84Q/84Q^ mouse slipping three times on the elevated beam assay.

### Swimming assay

**Figure 3.**
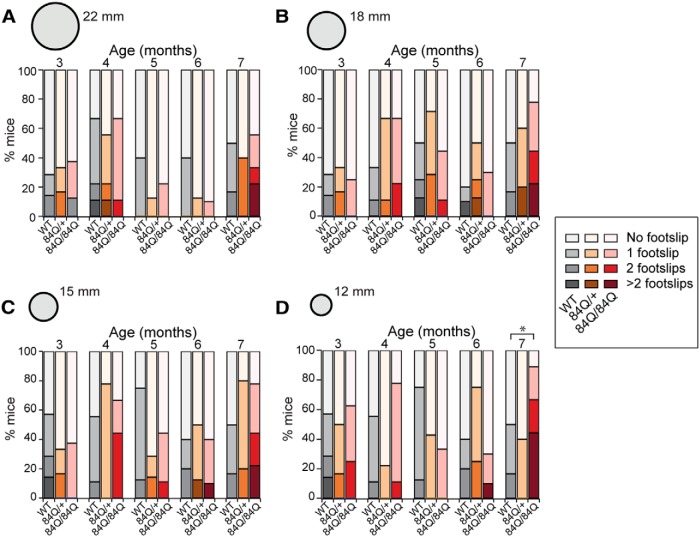
Increased footslips on narrow elevated beam at 7 months in SCA6^84Q/84Q^ mice. ***A*–*D***, The number of mice that display footslips (0 footslips = lightest color, >2 footslips = darkest color, and 1 and 2 footslips graded in between) when crossing beams for the following three genotypes: WT (grayscale) SCA6^84Q/+^ (orange scale), and SCA6^84Q/84Q^ mice (red scale); see legend on the right. No differences were seen across genotypes and age for the following: ***A***, 22-mm-diameter beam (*F*_(2,37)_ = 0.17; *p* = 0.85); ***B***,18-mm-diameter beam (*F*_(2,37)_ = 1.91; *p* = 0.16); ***C***, 15-mm-diameter beam (*F*_(2,37)_ = 0.65; *p* = 0.53); ***D***, a significant increase in the number of footslips was observed for the 12-mm-diameter beam at 7 months for SCA6^84Q/84Q^ mice (*F*_(2,37)_ = 4.19; *p* = 0.02). *p < 0.05; *p* > 0.05 where not indicated, one-way ANOVA followed by *post hoc* Tukey’s test; *N* = 8 − 10 SCA6^84Q/84Q^ mice depending on age, 5 − 9 SCA6^84Q/+^ mice, and 6 − 9 WT mice (consult Table 1 for sample size at each age).

Animals were trained to swim across a custom-built Plexiglas swimming tank (100 cm long by 6 cm wide) toward a dry, boxed-in escape platform (Carter et al., 1999; Fig. 3*A*; Movie 5-7). Bright light at the starting location was used as an aversive stimulus to encourage swimming across the tank. The mice were initially trained to swim across the swim tank toward the escape platform for two trials per day. D1 and D2 were considered training days, while testing days correspond to D3−D5. Mice were videotaped, and latency to traverse a 60 cm distance was recorded. The number of hindlimb kicks to cross the tank was counted during *post hoc* video analysis. See Movie 6,7; after the assay, mice were towel dried and monitored in their home cage for 20 min after the assay.

**Movie 5.** Swimming assay (top view). A 7-month-old SCA6^84Q/84Q^ mouse swims across the tank.

**Movie 6.** Swimming assay—SCA6^84Q/84Q^ mouse (side view). A 7-month-old SCA6^84Q/84Q^ mouse swims across the tank. Asterisks indicate right hindlimb kicks; 19 kicks were counted.

**Movie 7.** Swimming assay—WT mouse (side view). A 7-month-old WT mouse swims across the tank. Asterisks indicate right hindlimb kicks; 15 kicks were counted.

### Gait analysis

**Figure 4.**
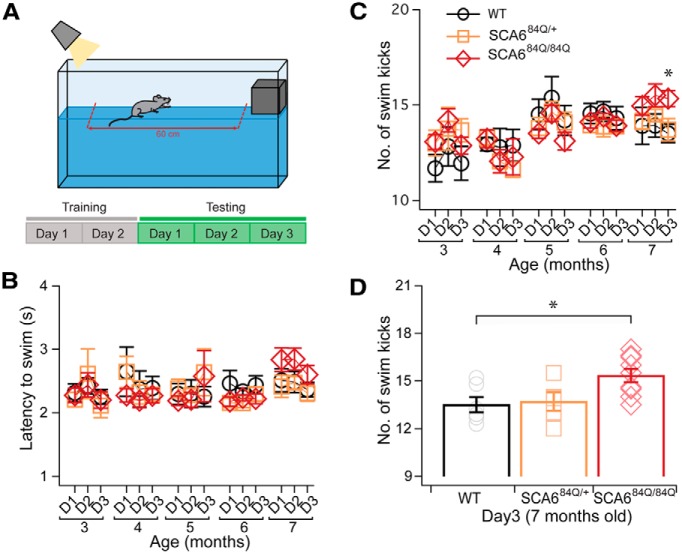
Swimming deficits at 7 months in SCA6^84Q/84Q^ mice. ***A***, Schematic showing the experimental design for the swimming assay. Mice were trained for 2 d and subsequently tested over 3 d (D1–D3 in ***B*** and ***C***). ***B***, No significant differences in swim latency were observed for mice across ages and genotypes (Age × Genotype: *F*_(8,105)_ = 1.85; *p* = 0.07; see Table 1 for *N* values). ***C***, In contrast with latency, there was an increase in the number of hindlimb kicks performed to cross the tank at 7 months of age in SCA6^84Q/84Q^ but not SCA6^84Q/+^ compared with WT mice (Age × Genotype × Days: *F*_(16,210)_ = 1.81; *p* = 0.03). *N* = 8 − 10 SCA6^84Q/84Q^ mice depending on age, 5 − 9 SCA6^84Q/+^ mice, and 6 − 9 WT mice (consult Table 1 for sample size at each age). ***D***, Summary data showing the number of kicks on Day 3 at 7 months old for the different genotypes. *p < 0.05 one-way ANOVA followed by Tukey’s *post hoc* test; N = 6 WT mice, 5 SCA6^84Q/+^ mice, and 9 SCA6^84Q/84Q^ mice.

**Figure 5.**
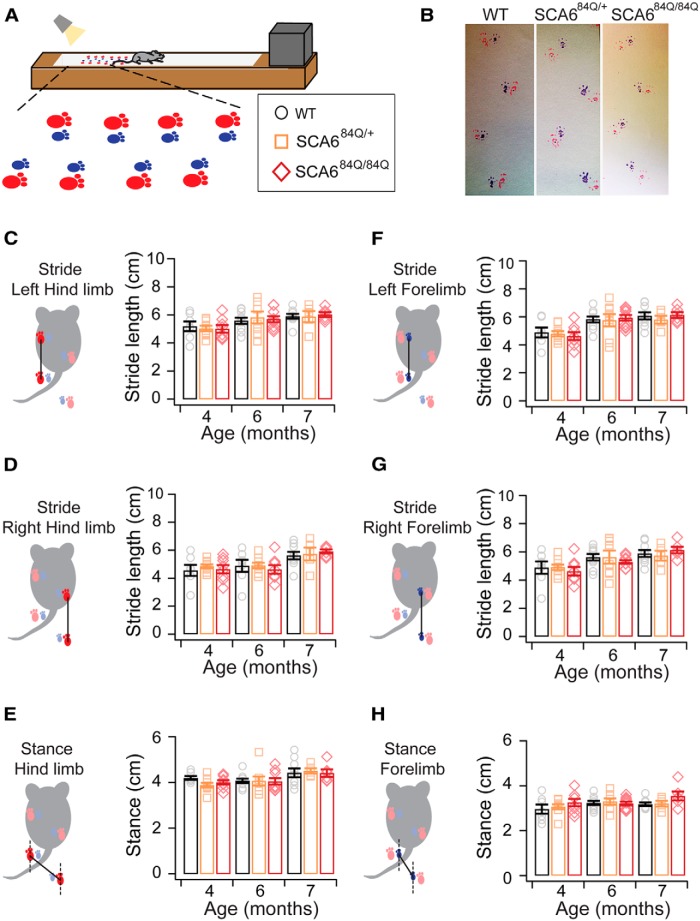
No abnormalities observed in gait in SCA6^84Q^ mice before or at the onset of motor coordination deficits. ***A***, Schematic of painted footprint experiment used to study gait: forelimbs were painted blue and hindlimbs were painted red. ***B***, Representative footprints from mice in each genotype reveal no significant differences in gait. ***C–H***, The distance between subsequent limb placements (stride length) at 4, 6, and 7 months of age were not significantly different across phenotypes for the following: ***C***, left hindlimb (*F*_(2,63)_ = 0.14; *p* = 0.87); ***D***, left forelimb (*F*_(2,63)_ = 0.25; *p* = 0.78); ***E***, right hindlimb (*F*_(2,63)_ = 0.46; *p* = 0.64); and, ***F***, right forelimb (*F*_(2,63)_ = 0.08; *p* = 0.91). Likewise, no significant differences were observed for stance (distance between left and right limb placements) of the following: ***G***, hindlimbs (*F*_(2,63)_ = 1.23; *p* = 0.30); and ***H***, forelimbs (*F*_(2,63)_ = 0.53; *p* = 0.60) at 4, 6, or 7 months. One-way ANOVA; *N* = 8 − 10 SCA6^84Q/84Q^ mice depending on age, 5 − 9 SCA6^84Q/+^ mice, and 6 − 9 WT mice (consult Table 1 for sample size at each age).

Gait was analyzed as previously described (Carter et al., 2001). The forelimbs and hindlimbs of each mouse were coated with distinct colors using nontoxic paint. Mice were prompted to walk across a white sheet affixed to an elevated platform (10 cm high by 10 cm wide) toward a custom-built dark escape box (Fig. 4*A*), leaving a trace of their paw prints on the sheet (Fig. 4*B*). Stride length (the distance between subsequent left and right forelimb and hindlimbs; Fig. 5*E*,*F*) and stance width (the distance between forelimbs and hindlimbs; Fig. 5*G*,*H*) were measured for four to six consecutive strides (measured between the centers of footprints). The coefficient of variation (CV) between stride lengths, as well as the degree of overlap between forelimb and hindlimb footprints were recorded from six consecutive strides. This assay was performed in a single trial on either D1 (at 1 and 2 years) or D5 of testing (at 4, 6, and 7 months).

### Immunocytochemistry

Mice were deeply anesthetized and perfused intracardially with 4% PFA (EMS). The brain was extracted and stored in PFA at 4°C for 24 h, then transferred to PBS with 0.5% sodium azide. The cerebellar vermis was sliced into 100-μm-thick parasagittal slices, and the striatum was sliced into 100-μm-thick coronal slices on a Leica Vibratome 3000 plus. Staining was performed in a blocking solution consisting of 5% BSA, 0.05% sodium azide, and 0.4% Triton X in 0.01 m PBS. The primary antibodies used were rabbit anti-Calbindin D-28k (Swant) at a dilution of 1:1000, and mouse-anti NeuN (MAB377, Millipore) tagged with Alexa Fluor 488 at a dilution of 1:500, and slices were incubated with this at room temperature on a rotary shaker at 70 rpm for 72 h. Slices were then rinsed three times in a solution of 0.4% Triton X in 0.01 m PBS, and for calbindin staining, a secondary antibody (Alexa Fluor 594 anti-rabbit, Jackson ImmunoResearch) was used at a dilution of 1:1000 in blocking solution and incubated for 90 min at room temperature while shaking. Sections were then rinsed and immediately mounted onto slides with Prolong gold antifade mounting solution (Life Technologies), and stored in the dark at 4°C. Slices were imaged with a custom-built two-photon microscope with a Ti:Sapphire laser (MaiTai, SpectraPhysics) tuned to 775 nm. Image acquisition was performed using ScanImage (Pologruto et al., 2003) running in Matlab (Mathworks). Purkinje cell numbers were counted in anterior (lobule 3) or posterior (lobule 9) vermis at a density of 100 μm Purkinje cells/layer, and molecular layer thickness was measured from the distance between the Purkinje cell layer at multiple evenly spaced locations in the lobule. Chemicals were purchased from Sigma unless otherwise indicated.

### Data analysis and statistics

All data were analyzed blind to genotype. For each behavioral assay, mouse performance was compared among the three genotypes using one-way ANOVA; when significance was found, this was followed by Tukey’s HSD *post hoc* test using JMP Software (SAS Institute). Purkinje cell density and molecular layer height were similarly compared with a one-way ANOVA followed by Tukey’s HSD *post hoc* test, and imaging data were acquired and analyzed blind to condition. Striatum cell counts were compared with Student’s *t* test. Data are reported as the mean ± SEM.

## Results

### Rapid onset of motor coordination abnormalities at 7 months old

An accelerating rotarod assay was used to test the motor coordination of mice at several postnatal ages, ranging from 3 to 7 months (Fig. 1*A*). The performance of mice on the rotarod was age dependent, since younger mice (3 and 4 months old) performed better than older mice (6 months old) on all three genotypes tested: SCA6^84Q/84Q^, SCA6^84Q/+^, and WT (age: *F*_(4,105)_ = 10.67; *p* < 0.0001; Fig. 1*B*). Similarly, we found that mice of all genotypes significantly increased their performance across days within an experimental age (comparing D5 to D1 × Age; *F*_(16,420)_ = 4.62; *p* < 0.0001 for 3, 4, 5, and 6 months; Fig. 1*B*) except at 7 months, where there was no significant increase in performance across days in any genotype (*p* = 0.57).

In agreement with an earlier study by Watase et al. (2008), we found that SCA6^84Q/84Q^ mice exhibited no motor abnormalities at 3 months, but displayed significant motor deficits compared with SCA6^84Q/+^ and WT mice at 7 months (Fig. 1*B*). We wondered whether the onset of motor deficits occurred between these ages, but found no differences in rotarod performance across genotypes at 4, 5, or 6 months (for sample size at each age, see Table 1; Fig. 1*B*), suggesting that the onset of motor abnormalities in SCA6^84Q/84Q^ mice does not occur earlier than 7 months, and that disease onset is relatively rapid between 6 and 7 months of age. Since some studies have found that other assays are more sensitive than rotarod for detecting early motor abnormalities (Stroobants et al., 2013; Larivière et al., 2015), we chose to test motor coordination with additional assays as well.

We next conducted an elevated beam assay to gain insight into motor coordination and balance in SCA6^84Q^ mice (Fig. 2*A*). Using beams of varying diameters, we measured the latency of the mice to cross the beam as an assay of motor coordination. Wide beams are typically easier for mice to walk across than narrow beams, and we reasoned that a range of beam sizes might capture subtle motor deficits of fine motor coordination that were not detectable with rotarod. In contrast to what we found for rotarod, older mice (6 months) performed better by crossing the beams faster than younger mice (3 months) for all genotypes tested, although this was true only for wide beams (Age: 22 mm beam, *F*_(4,105)_ = 4.72; *p* = 0.003; 18 mm beam, *F*_(4,105)_ = 2.36; *p* = 0.04; Fig. 2*B*,*C*), while no age dependence was observed for narrow-diameter beams (15 mm, *p* = 0.38; 12 mm, *p* = 0.14; Fig. 2*D*,*E*).

SCA6^84Q/84Q^ mice took longer to traverse the elevated beam at 7 months in comparison with WT and SCA6^84Q/+^ mice for the majority of beams used, while their performance was indistinguishable at earlier months (Fig. 2*B*–*E*). To look in more detail at the elevated beam phenotype, we measured the number of times the hindlimb feet of each mouse slipped during beam crossings (Movies 3, 4). For most trials, the majority of mice crossed the beam without any footslips, irrespective of genotype, age, or beam width (*p* > 0.05 for all beams and ages excluding the 12 mm beam at 7 months; Fig. 3). However, at 7 months, the majority of SCA6^84Q/84Q^ mice experienced footslips when crossing the narrowest beam, and this was significantly different from WT or SCA6^84Q/+^ mice (12 mm beam at 7 months: *F*_(2,37)_ = 4.19; *p* = 0.02; Fig. 3*D*). Thus, the increased latency to cross most beams for 7-month-old SCA6^84Q/84Q^ mice likely reflects motor coordination and/or balance abnormalities. SCA6^84Q/+^ mice showed no significant differences compared with WT mice for any age or beam diameter (*p* > 0.05; Figs. 2, 3). In summary, like rotarod, the elevated beam assay found motor coordination and balance abnormalities in SCA6^84Q/84Q^ mice at 7 months and no earlier, suggesting that the two assays are broadly similar in their ability to detect SCA6 motor abnormalities.

While rotarod and elevated beam are standard assays for motor coordination and balance deficits, we wanted to explore whether less standard motor assays might be useful to detect a motor phenotype in SCA6 mice. Swimming assays have been shown to detect subtle motor deficits at an earlier age than both rotarod and the elevated beam assay in Huntington’s disease (HD) mice (Carter et al., 1999), and we wondered whether this might be similar in SCA6. We used a swimming assay to further characterize motor performance in SCA6^84Q^ mice (Fig. 4*A*). There were no obvious visual differences in the coordination of the limbs when SCA6^84Q^ mice swam. Unlike with rotarod and the elevated beam assay, swimming performance showed no age-dependent differences across genotypes at all five ages tested, and the latency to cross the tank was not significantly different at any age across genotypes (3, 4, 5, 6, and 7 months; Age: *F*_(4,104)_ = 1.13; *p* = 0.35; Fig. 4*B*). Mice appear to rely mainly on hindlimbs for propulsion through the water when swimming (Movies 5-7). To determine whether there were changes in swimming performance that were not captured by measuring latency, we also counted the number of hindlimb swim kicks that were produced to traverse the tank. SCA6^84Q/+^ and WT mice had a similar number of kicks across all ages (*p* > 0.05; Fig. 4*C*). However, SCA6^84Q/84Q^ mice produced a small but significant increase in hindlimb kicks at 7 months on the third day of testing (Fig. 4*C*,*D*). These results strengthen our findings from rotarod and elevated beam assays that SCA6^84Q/84Q^ mice have normal motor ability and coordination up until 6 months, and significant motor deficits are detected 1 month later at 7 months of age, when more hindlimb kicks are required to traverse the swim tank.

### No gait abnormalities observed in SCA6^84Q/84Q^ mice

Gait abnormalities have been reported recently for presymptomatic SCA6 patients (Rochester et al., 2014) and have also been observed in an SCA6 mouse model with an even longer 118Q expansion repeat (Unno et al., 2012). While no differences in gait were observed by eye, we examined gait in SCA6^84Q/84Q^ mice using footprint analysis (Fig. 5*A*,*B*) and found no differences across genotypes for stride length (Fig. 5*C–F*) or stance width (Fig. 5*G*,*H*), and no differences across ages 3 − 7 months for all genotypes (SCA6^84Q/84Q^, SCA6^84Q/+^, and WT; *p* > 0.05 for all measurements).

To further test for possible changes in gait, we looked at the variance of stride lengths and paw overlap since stride lengths of mice are known to be very precise with minimal variation (low CV; Carter et al., 1999). We measured the CV of interstride distances from six consecutive strides to detect whether changes in this variance could be observed in SCA6^84Q^ mice. We found that the CV of hindlimb and forelimb strides was low and not significantly different across genotypes (hindlimb stride: Genotype, *F*_(2,63)_ = 0.27, *p* = 0.77; forelimb stride: Genotype, *F*_(2,63)_ = 0.10, *p* = 0.91; data not shown). We next compared the variance in paw overlap from forelimb and hindlimb paws, but found that, consistent with our other gait analyses, no significant differences in the CV of interpaw overlap was observed across genotypes (Genotype: *F*_(2,63)_ = 0.44; *p* = 0.56; data not shown). Furthermore, there were no significant age-dependent changes for any genotype (*p* > 0.05 for each measure; Table 2). These results suggest that SCA6^84Q/84Q^ mice have normal gait at 7 months despite exhibiting motor coordination deficits that were detected using rotarod (Fig. 1), elevated beam (Figs. 2, 3), and swimming assays (Fig. 4).

**Table 2:** Statistical table

Figure no.	Figure panel	Description	Test	Degrees of freedom	*F* value	*p* value	95% CI
1	*B*	Rotarod (3–7 months)—effect of age	ANOVA—fit model	4, 105	10.6731	<0.0001	
1	*B*	Rotarod (3–7 months)—effect of days × age	ANOVA—fit model	16, 420	4.6269	<0.0001	
1	*B*	Rotarod (3–7 months)—effect of genotype × age	ANOVA—fit model	8, 105	2.2818	0.0271	
1	*B*	Rotarod (3 months)—effect of genotype	One-way ANOVA	2, 39	2.5509	0.091	
1	*B*	Rotarod (4 months)—effect of genotype	One-way ANOVA	2, 51	0.2849	0.7533	
1	*B*	Rotarod (5 months)—effect of genotype	One-way ANOVA	2, 45	1.5495	0.2235	
1	*B*	Rotarod (6 months)—effect of genotype	One-way ANOVA	2, 53	0.6127	0.5457	
1	*B*	Rotarod (7 months)—effect of genotype	One-way ANOVA	2, 37	12.1937	<0.0001	
1	*B*	Rotarod (7 months)—WT × SCA6 84Q/84Q	Tukey-HSD			0.0004	14.17–52.89
1	*B*	Rotarod (7 months)—SCA6 84Q/84Q × SCA6 84Q/+	Tukey-HSD			0.0009	12.74–53.71
1	*B*	Rotarod (7 months)—WT × SCA6 84Q/+	Tukey-HSD			0.9994	−21.94 to 22.54
2	*B*	Balance beam latency, 22 mm (3–7 months)—effect of age	ANOVA—fit model	4, 105	4.7234	0.0026	
2	*B*	Balance beam latency, 22 mm (3–7 months)—effect of genotype × age	ANOVA—fit model	8, 105	2.0755	0.0446	
2	*B*	Balance beam latency, 22 mm (3 months)—effect of genotype	One-way ANOVA	2, 18	1.8397	0.1875	
2	*B*	Balance beam latency, 22 mm (4 months)—effect of genotype	One-way ANOVA	2, 24	1.3117	0.288	
2	*B*	Balance beam latency, 22 mm (5 months)—effect of genotype	One-way ANOVA	2, 21	1.8648	0.1797	
2	*B*	Balance beam latency, 22 mm (6 months)—effect of genotype	One-way ANOVA	2, 25	0.1779	0.838	
2	*B*	Balance beam latency, 22 mm (7 months)—effect of genotype	One-way ANOVA	2, 17	7.3589	0.005	
2	*B*	Balance beam latency, 22 mm (7 months)—WT × SCA6 84Q/84Q	Tukey-HSD			0.0101	0.78–5.88
2	*B*	Balance beam latency, 22 mm (7 months)—SCA6 84Q/84Q × SCA6 84Q/+	Tukey-HSD			0.021	0.46–5.86
2	*B*	Balance beam latency, 22 mm (7 months)—WT × SCA6 84Q/+	Tukey-HSD			0.987	−2.76 to 3.11
2	*C*	Balance beam latency, 18 mm (3–7 months)—effect of age	ANOVA—fit model	4, 105	2.3582	0.0398	
2	*C*	Balance beam latency, 18 mm (3–7 months)—effect of genotype × age	ANOVA—fit model	8, 105	3.0995	0.0035	
2	*C*	Balance beam latency, 18 mm (3 months)—effect of genotype	One-way ANOVA	2, 18	1.8068	0.1927	
2	*C*	Balance beam latency, 18 mm (4 months)—effect of genotype	One-way ANOVA	2, 24	0.0071	0.9929	
2	*C*	Balance beam latency, 18 mm (5 months)—effect of genotype	One-way ANOVA	2, 21	2.0014	0.1601	
2	*C*	Balance beam latency, 18 mm (6 months)—effect of genotype	One-way ANOVA	2, 25	0.7112	0.5007	
2	*C*	Balance beam latency, 18 mm (7 months)—effect of genotype	One-way ANOVA	2, 17	7.4618	0.0047	
2	*C*	Balance beam latency, 18 mm (7 months)—WT × SCA6 84Q/84Q	Tukey-HSD			0.0083	1.67–11.43
2	*C*	Balance beam latency, 18 mm (7 months)—SCA6 84Q/84Q × SCA6 84Q/+	Tukey-HSD			0.0243	0.73–11.06
2	*C*	Balance beam latency, 18 mm (7 months)—WT × SCA6 84Q/+	Tukey-HSD			0.9519	−4.95 to 6.26
2	*D*	Balance beam latency, 15 mm (3–7 months)—effect of age	ANOVA—fit model	4, 105	1.0691	0.3756	
2	*D*	Balance beam latency, 15 mm (3–7 months)—effect of genotype × age	ANOVA—fit model	8, 105	2.0276	0.05	
2	*D*	Balance beam latency, 15 mm (3 months)—effect of genotype	One-way ANOVA	2, 18	3.1614	0.0666	
2	*D*	Balance beam latency, 15 mm (4 months)—effect of genotype	One-way ANOVA	2, 24	0.5249	0.5982	
2	*D*	Balance beam latency, 15 mm (5 months)—effect of genotype	One-way ANOVA	2, 21	0.9172	0.4151	
2	*D*	Balance beam latency, 15 mm (6 months)—effect of genotype	One-way ANOVA	2, 25	0.4909	0.6178	
2	*D*	Balance beam latency, 15 mm (7 months)—effect of genotype	One-way ANOVA	2, 17	4.3447	0.0299	
2	*D*	Balance beam latency, 15 mm (7 months)—WT × SCA6 84Q/84Q	Tukey-HSD			0.0382	0.22–8.51
2	*D*	Balance beam latency, 15 mm (7 months)—SCA6 84Q/84Q × SCA6 84Q/+	Tukey-HSD			0.1167	−0.77 to 8.00
2	*D*	Balance beam latency, 15 mm (7 months)—WT × SCA6 84Q/+	Tukey-HSD			0.9143	−4.01 to 5.51
2	*E*	Balance beam latency, 12 mm (3–7 months)—effect of age	ANOVA—fit model	4, 105	1.7763	0.1391	
2	*E*	Balance beam latency, 12 mm (3–7 months)—effect of genotype × age	ANOVA—fit model	8, 105	2.1864	0.0342	
2	*E*	Balance beam latency, 12 mm (3 months)—effect of genotype	One-way ANOVA	2, 18	0.4304	0.6568	
2	*E*	Balance beam latency, 12 mm (4 months) effect of genotype	One-way ANOVA	2, 24	1.5008	0.2431	
2	*E*	Balance beam latency, 12 mm (5 months) effect of genotype	One-way ANOVA	2, 21	0.332	0.7212	
2	*E*	Balance beam latency, 12 mm (6 months) effect of genotype	One-way ANOVA	2, 25	0.4932	0.6165	
2	*E*	Balance beam latency, 12 mm (7 months) effect of genotype	One-way ANOVA	2, 17	5.2734	0.0165	
2	*E*	Balance beam latency, 12 mm (7 months)—WT × SCA6 84Q/84Q	Tukey-HSD			0.0427	0.16–10.23
2	*E*	Balance beam latency, 12 mm (7 months)—SCA6 84Q/84Q × SCA6 84Q/+	Tukey-HSD			0.0351	0.33–9.85
2	*E*	Balance beam latency, 12 mm (7 months)—WT × SCA6 84Q/+	Tukey-HSD			0.9987	−5.37 to 5.57
3	*A*	Balance beam footslips, 22 mm (3–7 months)—effect of age	ANOVA—fit model	4, 105	2.1833	0.0759	
3	*A*	Balance beam footslips, 22 mm (3–7 months)—effect of genotype × age	ANOVA—fit model	8, 105	0.5829	0.79	
3	*A*	Balance beam footslips, 22 mm (7 months)—effect of genotype	One-way ANOVA	2, 37	0.1683	0.8458	
3	*B*	Balance beam footslips, 18 mm (3–7 months)—effect of age	ANOVA—fit model	4, 105	1.589	0.1827	
3	*B*	Balance beam footslips, 18 mm (3–7 months)—effect of genotype × age	ANOVA—fit model	8, 105	0.6673	0.7191	
3	*B*	Balance beam footslips, 18 mm (7 months)—effect of genotype	One-way ANOVA	2, 37	1.9098	0.1624	
							
3	*C*	Balance beam footslips, 15 mm (3–7 months)—effect of age	ANOVA—fit model	4, 105	2.1859	0.0756	
3	*C*	Balance beam footslips,15 mm (3–7 months)—effect of genotype × age	ANOVA—fit model	8, 105	0.5983	0.7779	
3	*C*	Balance beam footslips, 15 mm (7 months)—effect of genotype	One-way ANOVA	2, 37	0.6498	0.528	
							
3	*D*	Balance beam footslips, 12 mm (3–7 months)—effect of age	ANOVA—fit model	4, 105	2.1259	0.0827	
3	*D*	Balance beam footslips, 12 mm (3–7 months)—effect of genotype × age	ANOVA—fit model	8, 105	2.1089	0.0412	
3	*D*	Balance beam footslips, 12 mm (3 months)—effect of genotype	One-way ANOVA	2, 39	0.1375	0.8719	
3	*D*	Balance beam footslips, 12 mm (4 months)—effect of genotype	One-way ANOVA	2, 51	0.2186	0.8044	
3	*D*	Balance beam footslips, 12 mm (5 months)—effect of genotype	One-way ANOVA	2, 45	1.4268	0.424	
3	*D*	Balance beam footslips, 12 mm (6 months)—effect of genotype	One-way ANOVA	2, 53	0.7188	0.492	
3	*D*	Balance beam footslips, 12 mm (7 months)—effect of genotype	One-way ANOVA	2, 37	4.1923	0.0229	
3	*D*	Balance beam footslips, 12 mm (7 months)—WT × SCA6 84Q/84Q	Tukey-HSD			0.0386	0.05–2.12
3	*D*	Balance beam footslips, 12 mm (7 months)—SCA6 84Q/84Q × SCA6 84Q/+	Tukey-HSD			0.0796	−0.10 to 2.10
3	*D*	Balance beam footslips, 12 mm (7 months)—WT × SCA6 84Q/+	Tukey-HSD			0.984	−1.11 to 1.27
4	*B*	Swimming latency (3–7 months)—effect of age	ANOVA—fit model	4, 105	1.1257	0.3484	
4	*B*	Swimming latency (3–7 months)—effect of genotype	ANOVA—fit model	2, 105	0.0301	0.9704	
4	*B*	Swimming latency (3–7 months)—effect of age × genotype	ANOVA—fit model	8, 105	1.8585	0.0744	
							
4	*C*	Swimming kicks (3–7 months)—effect of age	ANOVA—fit model	4, 105	1.8924	0.1421	
4	*C*	Swimming kicks (3–7 months)—effect of genotype	ANOVA—fit model	2, 105	1.4178	0.2468	
4	*C*	Swimming kicks (3–7 months)—effect of age × genotype	ANOVA—fit model	8, 105	1.6343	0.1238	
4	*C*	Swimming kicks (3–7 months)—effect of age × genotype × days	ANOVA—fit model	16, 210	1.812	0.0312	
4	*D*	Swimming kicks 7 months (Day 3 of testing)—SCA6 84Q/84Q × WT	Tukey-HSD			0.0375	0.07–6.71
4	*D*	Swimming kicks 7 months (Day 3 of testing)—SCA6 84Q/84Q × SCA6 84Q/+	Tukey-HSD			0.0439	0.09–6.78
4	*D*	Swimming kicks 7 months (Day 3 of testing)—SCA6 84Q/+ × WT	Tukey-HSD			>1	−3.68 to 3.94
5	*C*	Stride left hindlimb (4–7 months)—effect of genotype	ANOVA-fit model	2, 63	0.1432	0.8741	
5	*C*	Stride left hindlimb (4 months)—effect of genotype	One-way ANOVA	2, 21	0.1633	0.8504	
5	*C*	Stride left hindlimb (6 months)—effect of genotype	One-way ANOVA	2, 25	0.0946	0.91	
5	*C*	Stride left hindlimb (7 months)—effect of genotype	One-way ANOVA	2, 17	0.3729	0.6942	
5	*D*	Stride right hindlimb (4–7 months)—effect of genotype	ANOVA-fit model	2, 63	0.4552	0.6381	
5	*D*	Stride right hindlimb (4 months)—effect of genotype	One-way ANOVA	2, 21	0.1701	0.8447	
5	*D*	Stride right hindlimb (6 months)—effect of genotype	One-way ANOVA	2, 25	0.1729	0.8422	
5	*D*	Stride right hindlimb (7 months)—effect of genotype	One-way ANOVA	2, 17	0.0981	0.9071	
5	*E*	Stance hindlimb (4–7 months)—effect of genotype	ANOVA-fit model	2, 63	1.2341	0.2981	
5	*E*	Stance hindlimb (4 months)—effect of genotype	One-way ANOVA	2, 21	0.4725	0.6299	
5	*E*	Stance hindlimb (6 months)—effect of genotype	One-way ANOVA	2, 25	0.0238	0.9765	
5	*E*		One-way ANOVA	2, 17	0.5018	0.6141	
5	*F*	Stride left forelimb (4–7 months)—effect of genotype	ANOVA-fit model	2, 63	0.2511	0.7804	
5	*F*	Stride left forelimb (4 months)—effect of genotype	One-way ANOVA	2, 21	0.3767	0.6907	
5	*F*	Stride left forelimb (6 months)—effect ofgenotype	One-way ANOVA	2, 25	0.5051	0.6094	
5	*F*	Stride left forelimb (7 months)—effect of genotype	One-way ANOVA	2, 17	0.4583	0.6399	
5	*G*	Stride right forelimb (4–7 months)—effect of genotype	ANOVA-fit model	2, 63	0.0758	0.9124	
5	*G*	Stride right forelimb (4 months)—effect of genotype	One-way ANOVA	2, 21	0.2384	0.79	
5	*G*	Stride right forelimb (6 months)—effect of genotype	One-way ANOVA	2, 25	0.2074	0.7653	
5	*G*	Stride right forelimb (7 months)—effect of genotype	One-way ANOVA	2, 17	0.2588	0.775	
5	*H*	Stance forelimb (4–7 months)—effect of genotype	ANOVA-fit model	2, 63	0.5312	0.6042	
5	*H*	Stance forelimb (4 months)—effect of genotype	One-way ANOVA	2, 21	0.3596	0.7022	
5	*H*	Stance forelimb (6 months)—effect of genotype	One-way ANOVA	2, 25	1.2299	0.3094	
5	*H*	Stance forelimb (7 months)—effect of genotype	One-way ANOVA	2, 17	0.5272	0.5996	
6	*A*	Stride left hindlimb (1-2 years)—effect of genotype	ANOVA-fit model	1, 27	0.08	0.7654	
6	*A*	Stride left hindlimb (1 year)—effect of genotype	One-way ANOVA	1, 14	0.4616	0.5079	
6	*A*	Stride left hindlimb (2 years)—effect of genotype	One-way ANOVA	1, 13	0.1893	0.6707	
6	*B*	Stride right hindlimb (1-2 years)—effect of genotype	ANOVA-FIT model	1, 27	0.002	0.9704	
6	*B*	Stride right hindlimb (1 year)—effect of genotype	One-way ANOVA	1, 14	0.2275	0.6407	
6	*B*	Stride right hindlimb (2 years)—effect of genotype	One-way ANOVA	1, 13	0.3613	0.5581	
6	*C*	Stance hindlimb (1-2 years)—effect of genotype	ANOVA-fit model	1, 27	0.0001	0.9871	
6	*C*	Stance hindlimb (1 years)—effect of genotype	One-way ANOVA	1, 14	0.5178	0.4836	
6	*C*	Stance hindlimb (2 years)—effect of genotype	One-way ANOVA	1, 13	0.5202	0.4835	
6	*D*	Stride left forelimb (1-2 years)—effect of genotype	ANOVA-fit model	1, 27	0.0117	0.9045	
6	*D*	Stride left forelimb (1 year)—effect of genotype	One-way ANOVA	1, 14	0	1	
6	*D*	Stride left forelimb (2 years)—effect of genotype	One-way ANOVA	1, 13	0.0453	0.8348	
6	*E*	Stride right forelimb (1-2 years)—effect of genotype	ANOVA-fit model	1, 27	0.1049	0.7559	
6	*E*	Stride right forelimb (1 year)—effect of genotype	One-way ANOVA	1, 14	0.0692	0.7963	
6	*E*	Stride right forelimb (2 years)—effect of genotype	One-way ANOVA	1, 13	0.0308	0.8635	
6	*F*	Stance forelimb (1-2 years)—effect of genotype	ANOVA-fit model	1, 27	0.1742	0.6841	
6	*F*	Stance forelimb (1 year)—effect of genotype	One-way ANOVA	1, 14	0.5657	0.4644	
6	*F*	Stance forelimb (2 years)—effect of genotype	One-way ANOVA	1, 13	0.0059	0.94	
6	*E*	Rotarod (1 year)—effect of genotype	One-way ANOVA	1, 30	56.012	<0.0001	
6	*E*	Rotarod (1 year)—WT × SCA6 84Q/84Q	Tukey-HSD			<0.0001	44.07–77.15
6	*E*	Rotarod (2 years)—effect of genotype	One-way ANOVA	1, 28	33.6153	<0.0001	
6	*E*	Rotarod (2 years)—WT × SCA6 84Q/84Q	Tukey-HSD			<0.0001	19.35–40.48
7	*B*	Purkinje cell count/100 um (7 months)—effect of genotype	One-way ANOVA	1, 109	0.0023	0.9616	
7	*B*	Purkinje cell count/100 um (2 years)—effect of genotype	One-way ANOVA	1, 97	18.7953	<0.0001	
7	*B*	Purkinje cell count/100 um (2 years)—WT × SCA6 84Q/84Q	One-way ANOVA			<0.0001	0.58–1.57
7	*D*	Molecular layer length (7 months)—effect of genotype	One-way ANOVA	1, 203	0.7918	0.3746	
7	*D*	Molecular layer length (2 years)—effect of genotype	One-way ANOVA	1, 189	33.1151	<0.0001	
7	*D*	Molecular layer length (2 years)—WT × SCA6 84Q/84Q	One-way ANOVA			<0.0001	27.35–55.87
8	*B*	Density of striatal cells (7 months) WT × SCA6 84Q/84Q	Student's t-test	139 (t ratio)	0.3614	0.7184	-

**Figure 6.**
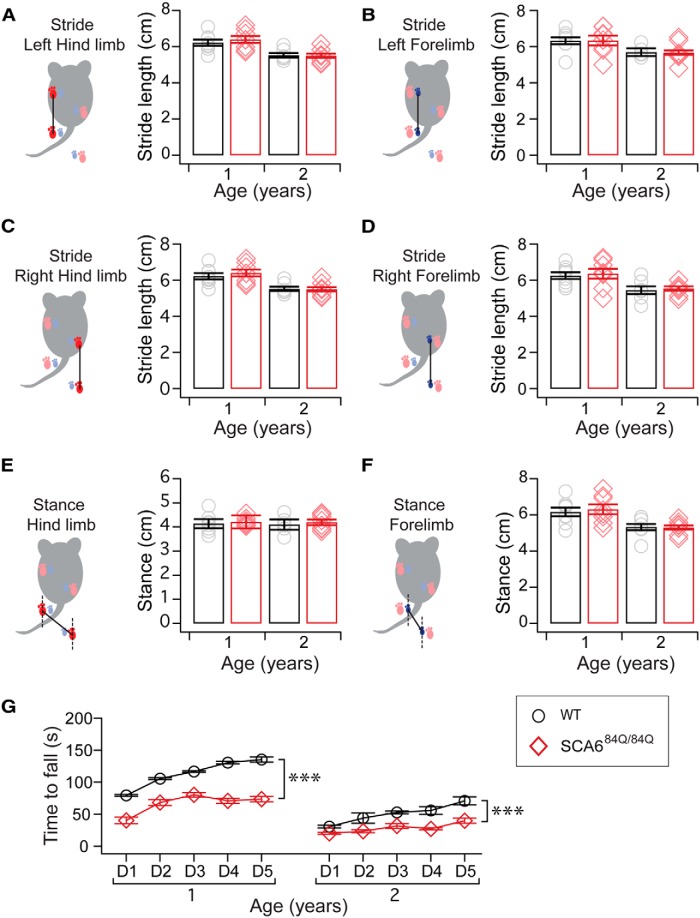
Disease progression marked by no gait abnormalities, but worsening motor coordination. ***A*–*F***, Gait was examined in aging animals that were 1 and 2 years old to determine whether differences in gait emerged as SCA6 progressed. The stride lengths at 1 and 2 years were not significantly different across SCA6^84Q/84Q^ and WT mice for the following: ***A***, left hindlimb (*F*_(1,27)_ = 0.08; *p* = 0.77); ***B***, left forelimb (*F*_(1,27)_ = 0.01; *p* = 0.90); ***C***, right hindlimb (*F*_(1,27)_ = 0.002; *p* = 0.97); and, ***D***, right forelimb stride lengths (*F*_(1,27)_ = 0.10; *p* = 0.76). Nor were significant differences observed for stance (distance between left and right limb placements) of the following: ***E***, hindlimbs (*F*_(1,27)_ = 0.0001; *p* = 0.99); and ***F***, forelimbs (*F*_(1,27)_ = 0.17; *p* = 0.68). ***G***, Motor coordination abnormalities worsened with age for 1- and 2-year-old mice on rotarod (1 year: Genotype, *F*_(1,30)_ = 56.01; *p* < 0.0001; 2 year: Genotype, *F*_(1,28)_ = 33.62; *p* < 0.0001). ***p < 0.001 one-way ANOVA followed by Tukey’s *post hoc* test; *N* = 8 WT and 8 SCA6^84Q/84Q^ 1-year-old mice; *n* = 7 WT and 8 SCA6^84Q/84Q^ 2-year-old mice.

We next tested gait in older SCA6^84Q/84Q^ mice to determine whether gait abnormalities emerged as the disease progressed, as has been observed in other mouse models (Unno et al., 2012). Using the same analyses as we performed at younger ages, we observed a difference comparing 1- and 2-year-old mice on several gait measurements across genotypes (Age: *p* < 0.005 for left and right forelimb and hindlimb stride, left and right stance, and CV of interpaw overlap; ANOVA followed by *post hoc* Tukey’s test; Fig. 6), although not all measures showed significant changes (no significant difference in the CV of left or right forelimb or hindlimb strides, *p* > 0.05; Fig. 6). These data suggest that there is a general age-related alteration in gait in aging mice. Surprisingly, there were no significant differences in any measure of gait in 1- or 2-year-old SCA6^84Q/84Q^ mice compared with age-matched WT mice (Fig. 6). This result strongly argues that gait abnormalities are not observed in SCA6^84Q/84Q^ mice throughout the majority of their lifespan, although they, like WT mice, experience aging-related gait alterations.

To assess disease progression in the same older mice, we examined rotarod performance in 1- and 2-year-old mice. Motor coordination abnormalities were observed in 1- and 2-year-old SCA6^84Q/84Q^ mice on rotarod (Fig. 6), and these deficits progressively worsened compared with deficits observed at 7 months old (Age × Genotype: *F*_(2,40)_ = 3.67; *p* = 0.034; Figs. 1, 6). Together, our data illustrate that motor coordination deficits have a rapid midlife onset in a narrow time window in SCA6^84Q/84Q^ mice, and that these symptoms progressively worsen without any alterations in gait.

### Late Purkinje cell degeneration in SCA6^84Q/84Q^ mice long after the onset of motor coordination deficits

**Figure 7.**
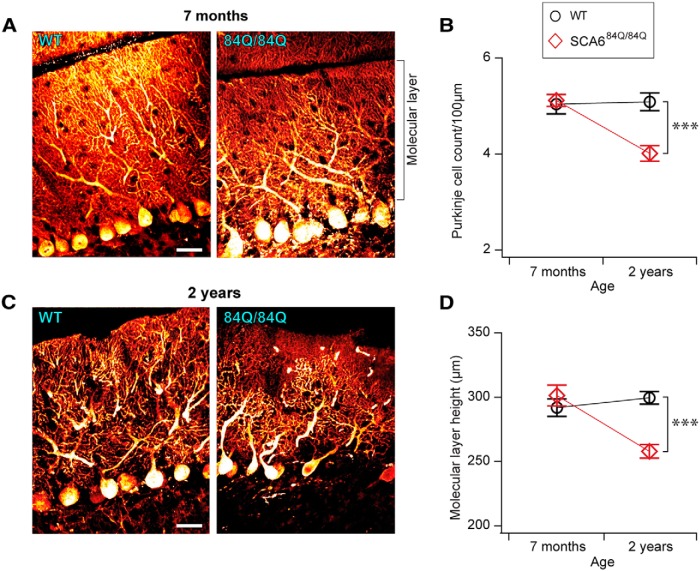
Purkinje cell degeneration is observed long after the onset of motor phenotype at 2 years in SCA6^84Q/84Q^ mice. ***A***, Representative images of calbindin-stained Purkinje cells from 7-month-old WT (left) and SCA6^84Q/84Q^ (right) mouse cerebellar slices. The height of the molecular layer is indicated. Scale bar, 20 μm. ***B***, Density of Purkinje cells in 7-month-old cerebellum is not significantly different in SCA6^84Q/84Q^ mice compared with WT mice (Genotype: *F*_(1,109)_ = 0.002, *p* = 0.96). However, reduced Purkinje cell density is observed at 2 years in SCA6^84Q/84Q^ mice (Genotype: *F*_(1,97)_ = 18.76, *p* = <0.0001; right). ***C***, Representative images of 2-year-old WT (left) and SCA6^84Q/84Q^ (right) Purkinje cells. Scale bar, 20 μm. ***D***, No significant difference in the Purkinje cell molecular layer is observed at 7 months in SCA6^84Q/84Q^ and WT mice (*F*_(1,203)_ = 0.79, *p* = 0.37; left), while molecular layer thickness is reduced at 2 years in SCA6^84Q/84Q^ mice compared with WT mice (*F*_(1,189)_ = 33.12, *p* < 0.0001; right). *N* = 3-4 animals for each genotype at each age; at least 10 mm of the Purkinje cell layer was measured for each comparison; one-way ANOVA with *post hoc* Tukey’s test. ***p < 0.0001, **p < 0.01, *p < 0.05; *p* > 0.05, where not indicated.

The transgenic SCA6^84Q/84Q^ mice that we used in this study have previously been reported to exhibit no Purkinje cells degeneration at 20 months old (Watase et al., 2008), which is in contrast to the degeneration observed early in 118Q hyperexpanded mice (Unno et al., 2012) and postmortem in human SCA6 patient data (Yang et al., 2000). We first examined Purkinje cell density in 7-month-old mice and found 5.2 ± 0.17 cells/100 μm Purkinje cell layer in WT mice, with no significant differences in SCA6^84Q/84Q^ mice (WT mice, 496 cells measured in 9.7 mm of the Purkinje cell layer from *N* = 3 animals; SCA6^84Q/84Q^ mice, 490 cells in 9.7 mm of the Purkinje cell layer from *N* = 3 animals; Fig. 7*A*,*B*), which is consistent with previous reports (Watase et al., 2008). We wondered whether subtle changes in Purkinje cell numbers or morphology might be restricted to only part of the cerebellum, since Purkinje cell degeneration has been reported to be more prevalent in anterior lobules of cerebellar vermis in some human patients (Gierga et al., 2009; Nanri et al., 2010). To address whether changes might be localized to subregions of the cerebellar vermis, we measured Purkinje cell density in anterior and posterior lobules, but observed no significant differences in both WT and SCA6^84Q/84Q^ mice (data not shown). To look in more detail at Purkinje cell morphology at disease onset, we measured the height of the molecular layer (Fig. 7*A*) as an estimate of the height of Purkinje cell dendritic trees and found no significant differences between WT and SCA6^84Q/84Q^ mice at 7 months (WT mice, 291.8 ± 6.8 μm; SCA6^84Q/84Q^ mice, 301.2 ± 8.1 μm; Fig. 7*A*,*D*). Thus, the onset of disease symptoms in SCA6^84Q^ mice is not associated with alterations in Purkinje cell number or gross dendritic morphology.

Since no changes in Purkinje cell numbers have been reported in 20-month-old SCA6^84Q/84Q^ mice (Watase et al., 2008), we looked for degeneration in older mice at 2 years. We observed no significant reduction in Purkinje cell density in 2-year-old WT mice compared with 7-month-old mice, suggesting that little degeneration has occurred at this age in WT mice (*p* = 0.99; Fig. 7*B*,*D*). However, we observed a reduction in the density of Purkinje cells in SCA6^84Q/84Q^ mice at 2 years compared with 7 months (*p* < 0.0001; Fig. 7*B*,*C*). Consistent with this, SCA6^84Q/84Q^ mice had ∼22% fewer Purkinje cells than their litter-matched WT siblings at 2 years (WT mice: 5.09 ± 0.18 cells/100 μm, 538 cells measured in 10.3 mm of Purkinje cell layer from *N* = 3 mice; SCA6^84Q/84Q^ mice: 4.01 ± 0.16 cells/100 μm, 385 cells in 9.8 mm of Purkinje cell layer from *N* = 3 mice; Fig. 7*D*). To address whether these changes were localized across the vermis, we compared the cell density in anterior and posterior lobules in SCA6^84Q/84Q^ mice, and found no significant differences at 2 years (anterior, 3.7 ± 0.19 cells/100 μm; posterior, 4.2 ± 0.25 cells/100 μm; *p* = 0.51). Rather, we observed a reduced Purkinje cell density in both anterior and posterior lobules of SCA6^84Q/84Q^ mice at 2 years compared with age- and litter-matched WT mice (anterior: *F*_(3,92)_ = 10.16; *p* < 0.0001; posterior: *F*_(3,106)_ = 3.37; *p* = 0.02; data not shown), in contrast to the predominantly anterior Purkinje cell degeneration observed in some human SCA6 patients (Gierga et al., 2009; Nanri et al., 2010).

Since Purkinje cell degeneration has been associated with both a reduction in cell number as well as structural changes in Purkinje cell dendrites (Yang et al., 2000), we looked at the height of the molecular layer as a readout of Purkinje cell dendritic alterations. We found that the mean molecular layer height in 2-year-old WT mice was 299.5 ± 4.9 μm, which was not significantly different from younger (7-month-old) WT mice (*p* = 0.72; Fig. 7*A*,*C*,*D*). Together with the cell count data for 2-year-old WT mice (Fig. 7*B*), this suggests that very little Purkinje cell degeneration has occurred in the vermis in aged WT mice. However, the average molecular layer thickness of SCA6^84Q/84Q^ mice at 2 years was 257.8 ± 5.3 μm, a ∼15% reduction from the molecular layer height in 7-month-old SCA6^84Q/84Q^ mice (*p* < 0.0001). We found that the molecular layer height in 2-year-old SCA6^84Q/84Q^ mice was significantly reduced compared with age-matched WT mice (Fig. 7*C*,*D*). Both our cell count and molecular layer data suggest that there is significant Purkinje cell degeneration by 2 years of age in SCA6^84Q/84Q^ mice.

**Figure 8.**
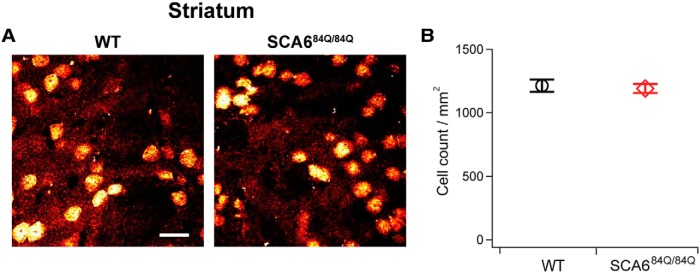
No loss of striatal neurons in SCA6^84Q/84Q^ mice accompanies the onset of motor coordination deficits at 7 months. ***A***, Representative images of NeuN-stained cells from 7-month-old WT (left) and SCA6^84Q/84Q^ (right) mouse striatum. Scale bar, 20 μm. ***B***, Density of striatal cells is not significantly different in SCA6^84Q/84Q^ compared with WT mice at 7 months (Student’s *t* test, *p* = 0.72).

Although SCA6 has been considered to be an example of a pure cerebellar ataxia (Solodkin and Gomez, 2012), noncerebellar symptoms are present in some patients, with up to 25% of affected individuals having signs of basal ganglia-related symptoms (Solodkin and Gomez, 2012). Since several recent studies have reported degeneration in the striatum of patients with other SCAs, including SCA2 and SCA3 (Schöls et al., 2015), and SCA17 (Brockmann et al., 2012), we looked at the number of striatal neurons in SCA6^84Q/84Q^ mice at 7 months to determine whether degeneration in the striatum was associated with the onset of motor abnormalities. We found that there were no significant differences between the density of cells in the striatum of SCA6^84Q/84Q^ and WT mice at 7 months (SCA6^84Q/84Q^ mice: 1191 ± 36 cells/mm^2^ striatum, total of 3332 cells counted from *N* = 4 mice; WT mice: 1212 ± 48 cells/mm^2^, total of 2586 cells counted from *N* = 3 mice; not significantly different, *p* = 0.72; Fig. 8).

## Discussion

We have performed an in-depth analysis of motor coordination and gait in a late-onset mouse model (84Q repeat length) of SCA6 in order to better understand the onset and progression of the SCA6 phenotype. We confirm that homozygous SCA6^84Q/84Q^ mice display motor coordination deficits at 7 months, and that deficits were detectable simultaneously in all motor coordination assays tested, including rotarod, elevated beam, and swimming. Prior to 7 months, the behavior of SCA6^84Q/84Q^, SCA6^84Q/+^, and WT mice were indistinguishable, arguing that motor coordination and performance in SCA6^84Q^ mice are normal prior to disease onset, and that motor deficits appear rapidly between 6 and 7 months in SCA6^84Q/84Q^ mice. Although Saegusa et al. (2007) report that heterozygous mice expressing a human-length (28Q) repeat show enhanced motor coordination compared with WT mice, we observe no significant differences between heterozygous SCA6^84Q/+^ and WT mice at any ages tested, which is in agreement with previous reports (Watase et al., 2008). Despite observing motor coordination deficits in 7-month-old SCA6^84Q/84Q^ mice, we observe no changes in any measures of gait, suggesting that this mouse model does not reproduce the progression of gait abnormalities typically observed for the human disease. Indeed, we were unable to detect changes in gait up to 2 years old, when motor coordination deficits had worsened, arguing that gait and motor coordination deficits can present independently. Finally, the onset of disease symptoms in SCA6 is not accompanied by morphological changes or the survival of cerebellar Purkinje cells, although Purkinje cell degeneration, reflecting both reductions in dendritic height and cell number, is observed nearly 1.5 years later in 2-year-old SCA6^84Q/84Q^ mice. The long time delay between disease onset and Purkinje cell degeneration suggests that Purkinje cell loss does not significantly contribute to early stages of SCA6 pathophysiology, and suggests that the potential for therapeutic intervention before cell death occurs might be a promising avenue of future study.

### Comparison of motor assays for detection of SCA6 and motor abnormalities

We studied a range of motor coordination assays in SCA6^84Q^ mice as it was unknown whether one motor assay would be more sensitive than others to subtle changes in motor coordination in SCA6. With rotarod, a mouse has the opportunity to slip only one time per trial, upon which it typically falls, which means that graded differences in motor performance may be underestimated. We reasoned that other motor coordination assays, like the elevated beam in which mice can slip multiple times during the completion of the task, might provide a more nuanced readout of motor deficits, as has been observed in some animal models of ataxia (Larivière et al., 2015), although there are animal models, like the SCA3 transgenic mouse, where deficits are detected with rotarod before elevated beam (Switonski et al., 2015). We also used a swimming assay that has been shown to detect motor abnormalities in an HD transgenic mouse earlier than the elevated beam (Carter et al., 1999). Since we observe similar results with three different motor coordination assays, we argue that these assays have comparable power to detect motor coordination deficit onset in SCA6. However, in our hands rotarod is simpler and easier to administer than the elevated beam or swimming assays, making it our preferred assay for SCA6 detection. It is possible, however, that even more sensitive assays exist to detect SCA6 motor abnormalities in rodents that we have not tested (Jarrahi et al., 2015; Vinueza Veloz et al., 2015).

We found robust motor deficits at 7 months without any concomitant changes in gait, and, indeed, gait abnormalities were not observed even in 2-year-old SCA6^84Q/84Q^ mice. This may at first appear surprising given that gait abnormalities are some of the first changes to be observed in SCA6 patients (Rochester et al., 2014), and gait abnormalities have been observed in an SCA6 mouse model with an even longer poly-Q expansion repeat (Unno et al., 2012). However, although motor coordination deficits and gait abnormalities often present together in mouse models (Chen et al., 2015; Swarnkar et al., 2015), this is not always the case: there are some ataxic models where gait abnormalities and motor coordination deficits are not temporally correlated (Clark et al., 1997; Simon et al., 2004; Larivière et al., 2015), while in other transgenic mouse models, gait abnormalities have even been shown to accompany enhanced performance on motor coordination assays (Nakatani et al., 2009; Piochon et al., 2014). The absence of gait abnormalities with motor coordination changes in SCA6^84Q/84Q^ mice highlights a limitation of this model in faithfully recapitulating human SCA6 symptoms (Rochester et al., 2014).

In addition to the reduced motor coordination observed with each motor assay for SCA6^84Q/84Q^ mice at 7 months, we also observed age-dependent changes in performance across genotypes that were strikingly different for the different motor assays we used: rotarod showed decreased motor performance with age, the elevated beam assay showed increased motor performance with age for wider beams, while swimming showed no apparent age-related differences in performance. While we and others have found decreased rotarod performance in aged WT mice (>18 months, Barreto et al., 2010; 2 years, Fig. 6*D*), which has been posited to arise because of neurodegeneration, we saw no significant reduction in WT Purkinje cell density at 2 years compared with 7 months (Fig. 7). In any case, degeneration cannot explain the age-dependent decreases in performance seen in 7-month-old mice of all genotypes with rotarod (Fig. 1). All three genotypes showed reduced performance with rotarod at 7 months compared with earlier performance; this suggests that there is a natural decline in performance at ∼7 months that is exacerbated in SCA6^84Q/84Q^ mice. Understanding the mechanism of aging-dependent decline at 7 months in all genotypes may provide insight into disease onset in SCA6.

Another difference between rotarod and the elevated beam assay is that for a given age, performance tends to improve on successive trials per day (data not shown) as well as over experimental days with rotarod (Fig. 1), while on the elevated beam mice tend to perform worse on the second day of training on most beam diameters (Fig. 2). In our experience, this day-on-day declining performance reflects at least in part the waning motivation of the mouse to cross the beam, which could confound the evaluation of motor coordination with this assay, while the day-on-day improvement observed with rotarod may involve cerebellar learning (Ly et al., 2013). For the swimming assay, there are few day-on-day or age-dependent changes in performance, suggesting that this assay may not be best suited to measure some aspects of motor performance. For these reasons, we find that rotarod is our best assay to detect motor learning alterations in mouse models of SCA6.

### Implications of SCA6^84Q^ Mouse Model for Human SCA6

While motor coordination abnormalities have been observed in SCA6^84Q/84Q^ mice at 7 months in a previous study (Watase et al., 2008), and because only a few time points were studied with a single motor assay in this previous report, the onset of disease symptoms was not well understood. Here we have characterized a rapid disease onset between 6 and 7 months that is detected with multiple behavioral assays, suggesting that disease onset is relatively strong since it can be detected by assays of varying sensitivity. This characterization of SCA6^84Q/84Q^ mice helps to strengthen this transgenic mouse as a model system for studying disease onset in SCA6. Our result of a narrow age of onset of disease symptoms is in contrast with observations in human patients, where individuals with a given repeat length can differ in age of onset by decades (van de Warrenburg et al., 2002). What might account for the discrepancy in disease onset variability between human patients and this transgenic mouse model? Transgenic SCA6^84Q^ mice are genetically homologous and live in a controlled environment, thus both the genetic and environmental diversity that exist in SCA6 patients may be absent in our study. Future enquiry is required to understand the contribution of epigenetic, environmental, and/or epistatic influences on SCA6 disease onset.

Since Purkinje cell loss is a common attribute of SCA6 (Yang et al., 2000), one of the limitations of the SCA6^84Q^ transgenic mouse model in the past has been the absence of reported Purkinje cell degeneration (Watase et al., 2008), unlike in other mouse models where Purkinje cell death is detected within weeks of disease onset (Unno et al., 2012). We observe Purkinje cell degeneration in 2-year-old SCA6^84Q/84Q^ mice, nearly 1.5 years after the onset of motor deficits, which argues that Purkinje cell death does not contribute to the early onset of motor abnormalities, and supports findings from other SCA6 mouse models suggesting that early symptoms of SCA6 arise from cellular alterations. Mechanisms that might contribute to the onset of motor dysfunction might be cellular inclusions in Purkinje cells (Watase et al., 2008; Mark et al., 2015), although Watase et al. (2008) show immunocytochemical evidence for these inclusions only at later ages (22 months), long after the onset of motor dysfunction in these mice. Other mechanisms contributing to early motor dysfunction might be due to a C-terminal fragment encoded by the *CACNA1A* gene, which may act as a transcription factor (Du et al., 2013) and/or may have deleterious action in the cytoplasm of Purkinje cells (Mark et al., 2015). Changes in synaptic input to Purkinje cells may also be involved (Mark et al., 2015). Our demonstration of Purkinje cell degeneration, a key feature of human SCA6 (Yang et al., 2000) in aged SCA6^84Q/84Q^ mice supports these mice as a good model system of SCA6. From a therapeutic perspective, these results are promising, as they suggest that a window for therapeutic intervention might exist where motor function could be ameliorated by the rescue of cellular abnormalities before cell death occurs. Rescue of motor function after cell death at later stages of the disease may be more challenging and may require different therapeutic approaches.

Several recent studies on SCAs have found that neural degeneration is not limited to cerebellum. For instance, the degeneration of neurons in the striatum has been observed in SCA2 and SCA3 (Schöls et al., 2015), and SCA17 (Brockmann et al., 2012). Interestingly, significant degeneration has been observed in the striatum without motor symptoms in both HD (Cowan and Raymond, 2006), and in several SCAs (Brockmann et al., 2012; Schöls et al., 2015), which made us wonder whether changes in the striatum might be involved in the onset of motor symptoms in SCA6. However, we found no significant cell loss in the stratum of SCA6^84Q/84Q^ mice at 7 months, suggesting that striatal degeneration is unlikely to contribute to early stages of disease onset in SCA6.

In summary, homozygous SCA6^84Q/84Q^ mice display motor coordination deficits that arise rapidly between 6 and 7 months of age without gait abnormalities or Purkinje cell degeneration. Motor coordination deficits progress, and Purkinje cell degeneration is observed in 2-year-old SCA6^84Q/84Q^ mice, confirming that these mice display this hallmark feature of human SCA6. The temporal lag between disease onset and neuronal degeneration argues that degeneration plays a role only in later stages of SCA6. These results are important as they suggest that a wide therapeutic window may exist after SCA6 disease onset before cell death occurs.

## Synthesis

The decision was a result of the Reviewing Editor Julie Andersen and the peer reviewers coming together and discussing their recommendations until a consensus was reached. A fact-based synthesis statement explaining their decision and outlining what is needed to prepare a revision is listed below.

The reviews are in good agreement and suggest that, while of potential interest to the field, additional information to address whether motor dysfunction at the 6-7 month time point observed in the 84Q expansion SCA6 model can be explained by alternative degenerative events is needed. The mutual decision reached by the reviewers' and reviewing editor is therefore a request for revision and re-review of the manuscript. Specific comments by the reviewers are included below.

Reviewer #1:

This work analyses the behavior and histological profile of a mouse model of spinocerebellar ataxia6 (SCA6) that has been previously published and is available at the Jackson Laboratories. The work is very well presented, with movies supporting the most important conclusions (although some editing is required, see further comments below). The most important results is linked to the observation that the mice show motor dysfunction (without gait abnormality) at 7 months of age. This correlates with the lack of Purkinje cell loss until 2 years of age.

There are a few concerns:

1- This reviewer does not completely understand the statement: "Purkinje cell degeneration was not observed at the age at which motor deficits were first detected, but significant Purkinje cell loss was observed in 2-year-old SCA684Q mice, arguing that neurodegeneration does not significantly contribute to the early stages of SCA6". This sentence is repeated a few times in the paper suggesting that the time-window between behavioral symptoms and histological pathology offers a good opportunity for therapy. Would it be possible to speculate or describe (based on the literature) the mechanisms inducing motor dysfunction in SCA6 unrelated to cerebellar pathology?

2-In Figure 2 (elevated beam test) and Figure 4C (swimming test, number of kicks), Q84/+ mice perform the same as Q84/Q84 mice. Are the Q84/+ statistically different compared to WT? Please address.

3-The calbindin staining for Q84/+ should be provided along with WT and Q84/Q84 images in Figure 7.

4-The presence of neuronal inclusions should be investigated by immunohistochemistry and/or biochemistry in different areas of the brain.

5-Motor dysfunction could be caused by alterations in striatum, loss of motoneurons in spinal cord or muscle atrophy. The reviewer is wondering if the authors have looked for any difference in their Q84/Q84 mice compared to controls.

6-In Figure 1, the animals present a maximum latency to fall of less than 180 seconds. In the Materials and Methods, the authors describe a protocol for rotarod of 10 minutes. Have the mice ever been able to run for the entire protocol? Please provide this information.

7-Movie 1: The entire video (accelerated) should be provided for one WT and one Q84/Q84 mouse to really appreciate the differences between the two genotypes.

8-Is it possible to highlight the swim kicks in Movie 4 and to present a video of one WT and one Q84/Q84 mouse?

Reviewer #2:

The manuscript entitled "Rapid onset of motor deficits in a knockin mouse model of spinocerebellar type 6 proceeds late cerebellar degeneration. This study carefully evaluates a knockin mouse model of SCA6 as a function of time. The authors generate littermate controls, SCA6 84Q/+ and SCA684Q/84Q. Then they test the mice in rotorod, elevated beam, swimming assay and gait. They also follow Purkinje cell count and molecular layer alterations. The study is sound and reports that motor changes precede loss of the Purkinje cells. Points that could be addressed are the following:

(1)Are the Purkinje cellular alterations the cause of the motor deficits in this model of SCA6? It is clear purkinje loss occurs much later in the lifespan of the mice. Have the authors looked at other regions of the brain that might influence motor coordination? In a related disease, HD, the loss of neurons may precede motor symptoms in humans so it would be useful to discuss this.

(2)The N for each group of mice seems low (Table 1). Could this impact why only at 7 months of age do the authors detect the motor deficit?

(3)Is the N for the Purkinje cell measurements indicated?

(4)Can the authors include RT-PCR or protein levels in the cerebellum of disease relevant message/proteins during this same time frame to give clues on how cellular changes occur relative to the motor deficits?

## Author Response

Below is a point-by-point response to the concerns raised by the editor and reviewer:

“I am pleased to inform you that your manuscript, “Rapid onset of motor deficits in a mouse model of spinocerebellar ataxia type 6 precedes late cerebellar degeneration,” has been judged potentially suitable for publication in eNeuro, provided appropriate revisions are made. The decision was a result of the reviewers and I coming together and discussing our recommendations until a consensus was reached. I have put together a fact-based synthesis statement explaining the decision and appended it to this email.”

We thank you and the reviewers for their time and helpful input.

“Synthesis Statement for Author (Required): The reviews are in good agreement and suggest that, while of potential interest to the field, additional information to address whether motor dysfunction at the 6-7 month time point observed in the 84Q expansion SCA6 model are due to degenerative events in, for example, the striatum are warranted. The mutual decision reached by the reviewers’ and reviewing editor is therefore a request for revision and re-review of the manuscript. Specific comments by the reviewers are included below.”

We have completed additional experiments as you and the reviewers suggested in the Synthesis Statement, to address whether degeneration in the striatum occurs at the time of the emergence of motor dysfunction in our SCA6 84Q model. We looked for changes in cell numbers at this age, using NeuN as a neuronal marker, and found that striatum cell counts were indistinguishable from WT controls, suggesting that degeneration of the striatum does not contribute to early pathogenicity in SCA6. We have included this data in a new figure (Figure 8), and have included further text in both the Results and Discussion sections.

Responding to Reviewer Number 1’s comments:

“This work analyses the behavior and histological profile of a mouse model of spinocerebellar ataxia6 (SCA6) that has been previously published and is available at the Jackson Laboratories. The work is very well presented, with movies supporting the most important conclusions (although some editing may be required). The most important results is linked to the observation that the mice show motor dysfunction (without gait abnormality) at 7 months of age. This correlates with the lack of Purkinje cell loss until 2 years of age.

We thank the reviewer for their helpful comments.

There are a few concerns:

1-This reviewer does not completely understand the statement: “Purkinje cell degeneration was not observed at the age at which motor deficits were first detected, but significant Purkinje cell loss was observed in 2-year-old SCA684Q mice, arguing that neurodegeneration does not significantly contribute to the early stages of SCA6. This sentence is repeated a few times in the paper suggesting that the time-window between behavioral symptoms and histological pathology offers a good opportunity for therapy. Would it be possible to speculate or describe (based on the literature) the mechanisms inducing motor dysfunction in SCA6 unrelated to cerebellar pathology?”

We have rewritten these sections to improve clarity, and have included an additional section in the Discussion where we speculate the mechanisms that might contribute to disease onset in SCA6.

“2-In Figure 2 (elevated beam test) and Figure 4C (swimming test, number of kicks), Q84/+ mice perform the same as Q84/Q84 mice. Are the Q84/+ statistically different compared to WT? Please address.”

The SCA6Q84/+ heterogeneous mice performance is indistinguishable from that of WT. We have clarified this in both the Results section and Figure Legends 2 and 4.

3-The calbindin staining for Q84/+ should be provided along with WT and Q84/Q84 images in Figure 7.

Because of the many months that we had to keep these mice in order to collect this data for this late time point, we unfortunately did not save litter-matched heterozygous mice for the 2-year time point, and thus cannot include Q84/+ staining in Figure 7.

4-The presence of neuronal inclusions should be investigated by immunohistochemistry and/or biochemistry in different areas of the brain.

We agree that the presence of neuronal inclusions in different brain areas is of interest to our study of SCA6. Watase and colleagues (2008) have reported neuronal inclusions with immunocytochemistry in Purkinje cells from the 84Q/84Q mouse model of SCA6 at 22 months old, which we have highlighted more clearly in the Discussion. This is ~ 15 months after we have observed motor deficits for the first time in the SCA684Q/84Q mouse. Therefore, we speculate that neuronal inclusions in Purkinje cells may not play a role in early pathophysiology of SCA6. While additional work on neuronal inclusions in other brain regions would be of interest, it is beyond the scope of this paper, and would be best addressed in an in-depth follow-up study.

5-Motor dysfunction could be caused by alterations in striatum, loss of motoneurons in spinal cord or muscle atrophy. The reviewer is wondering if the authors have looked for any difference in their Q84/Q84 mice compared to controls.

We agree with the reviewer that these are interesting questions. As mentioned above, we have completed additional experiments to address whether degeneration in the striatum occurs at the time of the emergence of motor dysfunction in our SCA684Q model, and find no significant difference between striatal neurons counts in 84Q/84Q and WT mice. Changes in the spinal cord or muscle atrophy would be very interesting to address as well, although human SCA6 patients have not been reported to have spinal cord or muscle abnormalities (Solodkin and Gomez, 2012). We have not addressed these questions experimentally as they are outside the scope of this paper, and would be better suited for an in-depth follow-up study.

6-In Figure 1, the animals present a maximum latency to fall of less than 180 seconds. In the Materials and Methods, the authors describe a protocol for rotarod of 10 minutes. Have the mice ever been able to run for the entire protocol? Please provide this information.

Neither SCA6 84Q/84Q nor WT mice were able to run for the entire 10-minute-long protocol. This may be due to the ages of the mice studied, or may be a particularity of the mouse strains that we study. If we compare our result to a previous study (Watase et al., 2008) using the same 84Q/84Q mice, it appears that both WT and 84Q/84Q mice stay on the Rotarod for approximately the same amount of time in our study. Importantly, the relative difference between 84Q/84Q and WT performance is similar in our study and that of Watase et al (2008). It appears, as well, that in this previous study (Watase et al., 2008), the WT and 84Q/84Q mice were unable to stay on the Rotarod for the full 10 minutes of testing, just like in our study.

Brown and Wong (2007) have compared Rotarod performance across different mouse species, and see significant differences in performance. The performance of our WT mice falls in the middle of the range of different mouse species. We have preliminary data suggesting that prior experience with Rotarod, even months earlier, can significantly improve Rotarod performance in wildtype mice (unpublished results). Since we did not want to miss subtle changes that might be altered from prior training, we did all our experiments in naive animals. We also performed all experiments in littermatched controls to account for any variability that might arise from genetic drift over generations.

7-Movie 1: The entire video (accelerated) should be provided for one WT and one Q84/Q84 mouse to really appreciate the differences between the two genotypes.

This is a good idea. We have included the entire video, accelerated, for one WT and one 84Q/84Q mouse, as the reviewer requests (Movie 2).

8-Is it possible to highlight the swim kicks in Movie 4 and to present a video of one WT and one Q84/Q84 mouse?

We have included a video of one WT and one 84Q/84Q mouse highlighting the difference in swim kicks for the reviewer to observe (Movie 5 and 6). We feel that it is rather difficult to identify the difference in swim kicks across genotypes, because although significant, it is not a large difference, and it is difficult to discern swim kicks to an untrained eye. We have tried to help this visualization by indicating the swim kicks with asterisks.

Reviewer #2: The manuscript entitled “Rapid onset of motor deficits in a knockin mouse model of spinocerebellar type 6 proceeds late cerebellar degeneration. This study carefully evaluates a knockin mouse model of SCA6 as a function of time. The authors generate littermate controls, SCA6 84Q/+ and SCA684Q/84Q. Then they test the mice in rotorod, elevated beam, swimming assay and gait. They also follow Purkinje cell count and molecular layer alterations. The study is sound and reports that motor changes precede loss of the Purkinje cells. Points that could be addressed are the following:

We thank the reviewer for their helpful comments.

(1) Are the Purkinje cellular alterations the cause of the motor deficits in this model of SCA6? It is clear purkinje loss occurs much later in the lifespan of the mice. Have the authors looked at other regions of the brain that might influence motor coordination? In a related disease, HD, the loss of neurons may precede motor symptoms in humans so it would be useful to discuss this.

This is a useful point. As mentioned above, we have completed additional experiments to address whether degeneration in the striatum occurs at the time of the emergence of motor dysfunction in our SCA6 model, and find no significant difference between striatal neurons counts between 84Q/84Q and WT mice. This data has been included in the new Fig. 8.

(2) The N for each group of mice seems low (Table 1). Could this impact why only at 7 months of age do the authors detect the motor deficit?

This is an important point. Because of the many months it takes to obtain animals of the right age, the overall N in each group was limited. The data presented in our study is thus hard won. The N’s should, however, be large enough to detect appreciable differences. To be rigorous, we performed experiments at each age group in naive mice from litter-matched controls, which also limited the N’s that we had for these studies. Furthermore, it has previously been shown for heterozygous 84Q/+ mice that there is no motor phenotype at 7 months (Watase et al., 2008). Thus, for the few cases where the N was lower for heterozygous animals, we felt that this was unlikely to impact the findings if we found changes that were consistent with this earlier report, as we did: no motor coordination phenotype in heterozygous mice, even at 7 months.

(3) Is the N for the Purkinje cell measurements indicated?

Thanks for picking up on this omission. The N for Purkinje cell measurements (number of cells counted in total mm Purkinje cell layer) has been added to the main text Results section.

(4) Can the authors include RT-PCR or protein levels in the cerebellum of disease relevant message/proteins during this same time frame to give clues on how cellular changes occur relative to the motor deficits?

This is a very interesting suggestion that merits further study, but it lies outside the scope of this paper, and is more suited to an in-depth follow-up study. We are in fact working on another study already, and we will attempt to work this and the above suggestions into this new study.
